# Bivariate genomic analysis identifies a hidden locus associated with bacteria hypersensitive response in *Arabidopsis thaliana*

**DOI:** 10.1038/srep45281

**Published:** 2017-03-24

**Authors:** Biao Wang, Zhuocheng Li, Weilin Xu, Xiao Feng, Qianhui Wan, Yanjun Zan, Sitong Sheng, Xia Shen

**Affiliations:** 1 Research Center of Precision Medicine, College of Life Sciences and Oceanography, Key laboratory of Optoelectronic Devices and System of Ministry of Education and Guangdong Province, College of Optoelectronic Engineering, Shenzhen University, Shenzhen, China; 2 Department of Medical Epidemiology and Biostatistics, Karolinska Institutet, Stockholm, Sweden; 3 Shenzhen Second People’s Hospital, the First Affiliated Hospital of Shenzhen University, Shenzhen, China; 4 State Key Laboratory of Biocontrol, School of Life Sciences, Sun Yat-sen University, Guangzhou, China; 5 Department of Medical Biochemistry and Microbiology, Uppsala University, Uppsala, Sweden; 6 HYK High-throughput Biotechnology Institute, Shenzhen, China; 7 Usher Institute of Population Health Sciences and Informatics, University of Edinburgh, Edinburgh, United Kingdom

## Abstract

Multi-phenotype analysis has drawn increasing attention to high-throughput genomic studies, whereas only a few applications have justified the use of multivariate techniques. We applied a recently developed multi-trait analysis method on a small set of bacteria hypersensitive response phenotypes and identified a single novel locus missed by conventional single-trait genome-wide association studies. The detected locus harbors a minor allele that elevates the risk of leaf collapse response to the injection of *avrRpm1*-modified *Pseudomonas syringae (P* = 1.66e-08). Candidate gene *AT3G32930* with in the detected region and its co-expressed genes showed significantly reduced expression after *P. syringae* interference. Our results again emphasize that multi-trait analysis should not be neglected in association studies, as the power of specific multi-trait genotype-phenotype maps might only be tractable when jointly considering multiple phenotypes.

In recent years, genome-wide association studies (GWAS) have been widely adapted into species other than human, e.g. model species such as *Arabidopsis thaliana*^1^,^2^,^3^,^4^ and *Drosophila melanogaster*^5^,^6^. Substantial gene discovery power has been observed in such model species especially with inbred lines, as noise in the data can be well controlled by designed experimental conditions, sample repeats and absence of heterozygote individuals. This provides us an opportunity to unravel genetic architecture of complex traits even using a much smaller population compared to humans.

Despite the fact that different multivariate methods have been proposed for GWAS of more than one phenotype, few studies in model species have been conducted using multivariate techniques^7^,^8^. This can be due to the complexity of interpreting multivariate statistical analysis results and the lack of user-friendly computational tools. With our experience in re-analyzing *A. thaliana* genomic data from different sources, we foresaw the potential power gain when applying multivariate analysis. We have recently developed a fast multivariate GWAS tool that directly works with single-trait GWAS summary-level data^9^, thus combining with conventional mixed model analysis, we can conduct multivariate GWAS in highly structured populations such as *A. thaliana*.

In order to deliver easy-to-interpret analysis, in this paper we focus on bivariate GWAS, i.e. limiting the dimension of multiple phenotypes to only two. We perform the bivariate GWA analysis on the previously published data of bacteria hypersensitive traits, because of their high statistical and biological relatedness that may potentially lead to power of joint analysis, and of the availability of the gene expression database with particular bacteria treatments that allows us to verify plausible candidate genes.

In the rest of the paper, we demonstrate how a locus, previously hidden in conventional single-trait analysis, can now be detected via our multivariate technique. With statistical interpretation and bioinformatic justification, we aim to provide a clear example of power gain and emphasize the importance of joint analyzing correlated phenotypes in different inbred populations.

## Results

We first downloaded the phenotypic and genotypic data published by ref. 1, where the four bacteria hypersensitive response phenotypes were previously published by ref. 10, i.e. *avrPphB* (n = 90), *avrRpm1* (n = 84), *avrB* (n = 87), *avrRpt2* (n = 89), corresponding to leaf collapse response to the injection of transformed strains of *Pseudomonas syringae* for the four pathogenic avirulence (*avr*) genes. We performed single-trait GWA analysis for each of the four traits based on a linear mixed model to adjust for population stratification of the *A. thaliana* population. Variants with minor allele frequencies (MAF) less than 0.05 were excluded, and subsequent genomic control^11^ was applied to stringently control the global false positive rate.

**Figure 1:**
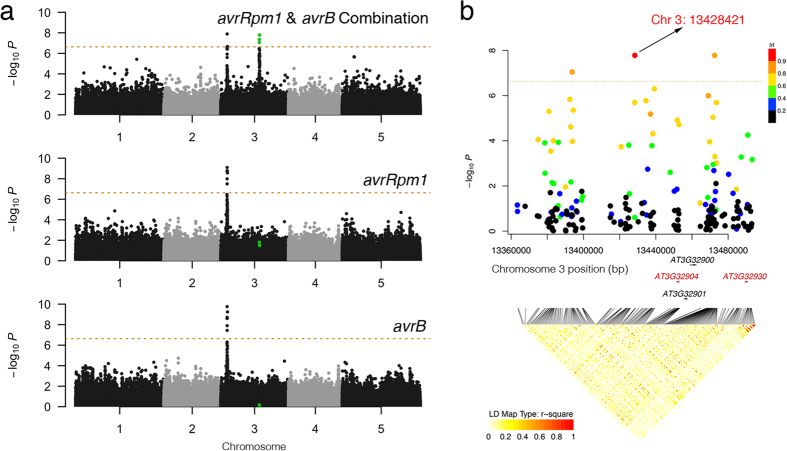
Bivariate genome-wide association analysis of two hypersensitive response traits *avrRpm1* and *avrB*. (**a**) Manhattan plots comparison of bivariate and univariate analysis results, where the novel variants only discoverable when combining two phenotypes are shown in green. The horizontal dashed line represents a 5% Bonferroni-corrected genome-wide significant threshold for 214 051 tests. (**b**) The LD block of the novel locus detected using bivariate analysis. Three plausible candidate genes are marked. r: linkage disequilibrium measured as correlation coefficient between the top variant and each variant in the region.

We considered all pairwise combination of the four hypersensitive response phenotypes to boost discovery power of functional loci. Using our recently developed multi-trait GWAS method, we can detour complicated multivariate modeling for binary data using single-trait GWAS summary statistics, i.e. genome-wide effects estimates (log odds-ratios) and standard errors for each of the hypersensitive response traits. Out of the six pairs of traits, one single novel locus reached genome-wide significance threshold for the combination of the two traits *avrRpm1* and *avrB* (Fig. 1a). The association LD block covers about a 130 kb region on chromosome 3 (Fig. 1b), with two top variants at 13 428 421 bp and 13 472 547 bp (bivariate *P* = 1.66e-08 for each variant).

**Figure 2:**
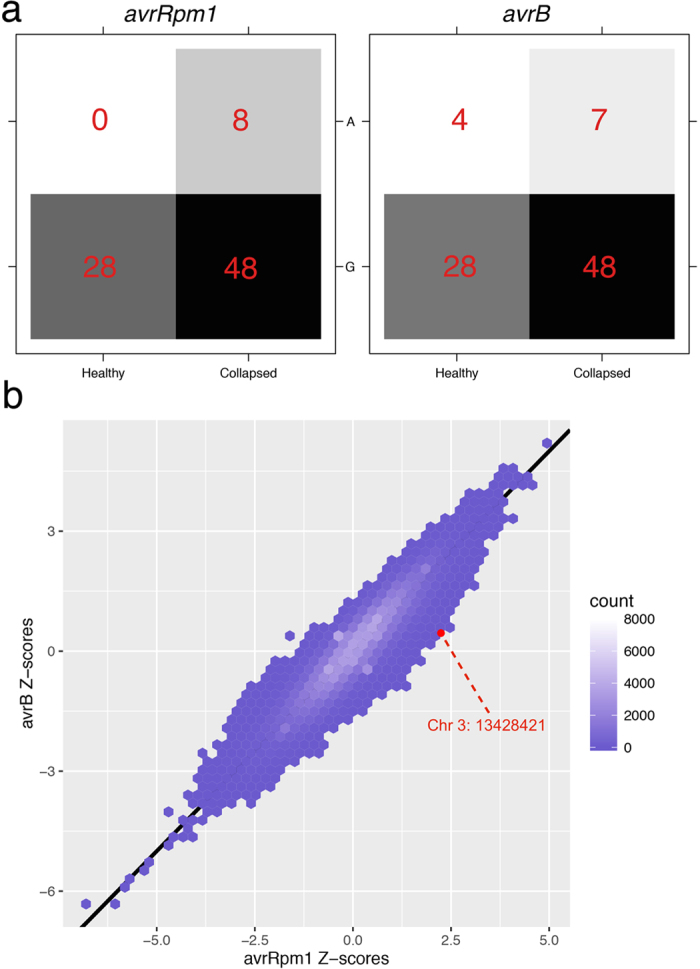
Bivariate statistical significance of the newly detected locus. (**a**) Genotype-phenotype map of the top variant for both phenotypes as 2 × 2 contingency tables. (**b**) Scatter plot comparing all Z-scores of the two traits across the genome. The top variant of the novel locus is marked on the edge of the empirical bivariate normal distribution.

The locus was missed in the single-trait GWA analysis of *avrRpm1* (OR = 1.23, *P* = 0.032) and that of *avrB* (OR = 1.01, *P* = 0.950). The statistical significance can only be identified when considering the joint statistic of the two phenotypes. According to the genome-wide Z-scores, these two traits are phenotypically highly correlated (estimated phenotypic correlation = 0.95 on the liability scale). In fact, the two *P. syringae avr* genes share the same host resistance gene (a.k.a. R-gene) in the *A. thaliana* genome, i.e. the *RPM1* gene^12^, which is why the *RPM1* locus on chromosome 3 is strongly associated with both traits (Fig. 1a). Namely, the two traits are biologically and statistically similar. However, the observed odds ratios on the two traits showed sufficient deviation that led to bivariate significance (Fig. 2), i.e. for such highly correlated phenotypes, we do not expect a big difference in terms of odds ratios at any SNP marker. Such a signal rejects the null hypothesis of no genetic effect exist at the detected locus, suggesting a causal effect that may be only on *avrRpm1* instead of *avrB*.

**Figure 3:**
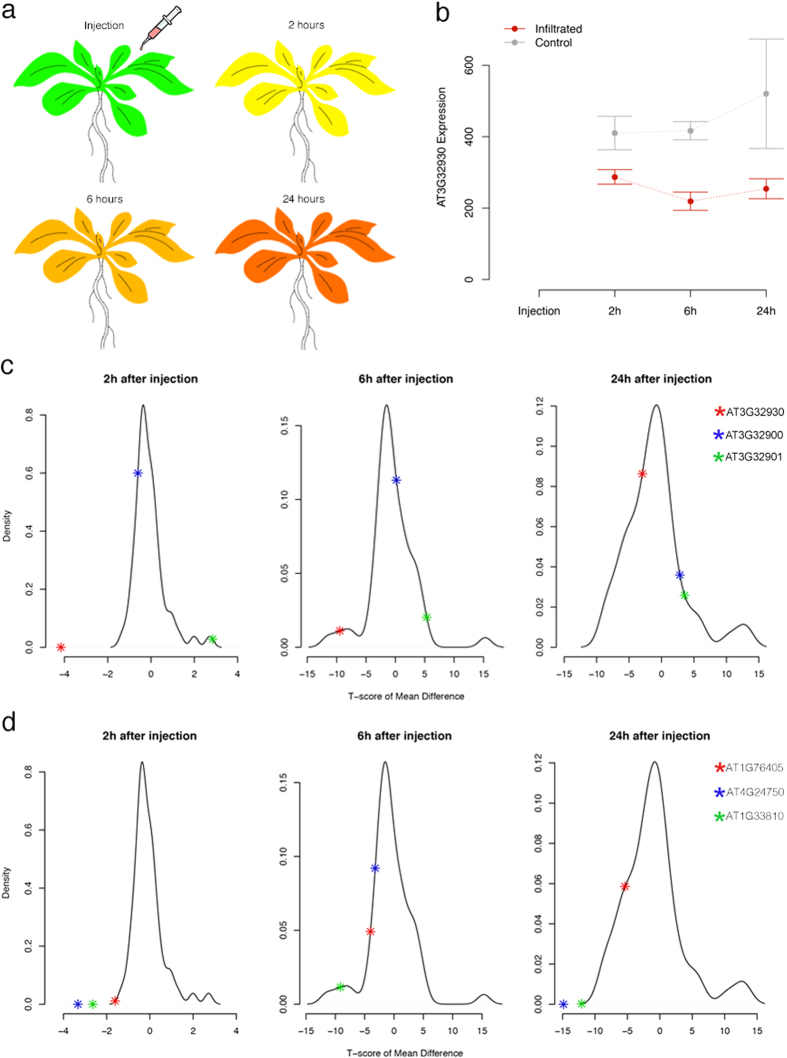
Expression of *AT3G32930* significantly decreases after injection of *P. syringae* pv. *avrRpm1*. (**a**) Colored illustration of the amount of gene expression at different time points after injection. (**b**) Comparison of gene expression measurements between infiltrated plants (avirulent 10e8 cfu/ml pv. *avrRpm1*) and control plants (10 mM MgCl_2_) at different time points. (**c**) Comparing gene expression change of three candidate genes at each time point with that of 50 randomly selected genes in the genome as positive controls. The expression difference between infiltrated and control plants is scored as a t-statistics of the mean difference. (**d**) Comparing gene expression change of three co-expressed genes of *AT3G32930* at each time point with that of 50 randomly selected genes in the genome as positive controls.

Although we lacked a set of independently measured data to replicate the genotype-phenotype map for the two hypersensitive response traits, we analyzed available gene expression data to identify plausible candidate genes. We downloaded the transcriptomic data of 144*A. thaliana* accessions and tried to screen the detected locus for cis-regulated eQTL^13^. Unfortunately, this locus is located in a low-expression region where none of the genes nearby shows reliable transcripts measurements after quality control (see Materials & Methods). We then hypothesised that the functional gene underlying such hypersensitive response traits might only be over- or under-expressed under treatment or in a specific genetic background. Therefore, we tested the first hypothesis using data from Arabidopsis eFP Browser^14^, where means and standard deviations of gene expression levels at different time points after *avrRmp1* modified *P. syringae* treatment are extracted for the Columbia-0 accession. Three genes *AT3G32900, AT3G32901* and *AT3G32930* showed significant expression difference compared to the negative control plants at certain time points after injection. According to the genome annotation, *AT3G32900* and *AT3G32901* appeared to be pseudogenes, so their expression changes after injection might be caused by a global effect on the whole transcriptome rather than true positives, e.g. the probes actually captured the homologs of these pseudogenes. Thus, as a positive control group, we randomly selected 50 genes across the genome to obtain a global empirical distribution of gene expression change after injection. For each gene at each time point after injection, we calculate the T-score testing the mean difference in expression between the infiltrated and control groups. We found the expression difference of *AT3G32930* is significantly different from that of the other genes (empirical *P* = 0.02) only at the time point of 2 hours after injection (Fig. 3b,c).

As functional study of the candidate gene *AT3G32930* is lacking, we used STRING^15^ (Search Tool for the Retrieval of Interacting Genes/Proteins, string-db.org) to search for interacting genes with *AT3G32930*. Four co-expressed genes *AT4G24750 (STR11*), *AT5G21920 (YLMG2*), *AT1G33810*, and *AT1G76405* with *AT3G32930* were detected, where three (except for *YLMG2*) have database records in Arabidopsis eFP Browser for the same *P. syringae* interference. All the three co-expressed genes, just as *AT3G32930* does, have significantly reduced expression compared to the random set of genes in the genome (empirical *P* < 0.02) at 2 hours after injection. The two genes *AT4G24750* and *AT1G33810* also have significantly reduced expression level at 24 hours after injection. This suggests that the co-expressed gene network containing the candidate gene *AT3G32930* reacts relatively stronger to the interference of *P. syringae* pv. *avrRpm1*.

In addition, we downloaded the whole-genome sequence data of 1 135*A. thaliana* accessions^4^ and extracted all the sequence variants that had LD r^2^ > 0.50 within 65 kb distance upstream and downstream of the variants with the highest significance, covering the LD block at the detected locus. These variants were passed onto Ensembl variant effect predictor (http://www.ensembl.org/Tools/VEP) for evaluation of their allelic substitution effects on the corresponding proteins. Two missense mutations, one at 13 451 746 bp changing codon TAT (for amino acid tyrosine) to GAT (for aspartic acid) and the other at 13 451 920 bp changing codon GAA (for glutamic acid) to AAA (for amino acid lysine), located inside *AT3G32904* were detected. As a hypothetical protein, no specific functional information of *AT3G32904* is available, for which no significant gene expression was detected in both our expression datasets, either. Given the above gene expression analysis, it is more likely that *AT3G32930* is the driver of this specific association signal.

## Discussion

Using our recently developed multi-trait analysis technique, we re-analyzed a GWAS dataset of four correlated bacteria hypersensitive response phenotypes in *A. thaliana*. A novel locus, hidden in the original single-trait analysis, was detected, where the rare allele showed significantly higher response to *P. syringae* pv. *avrRpm1* injection.

Analysis of available gene expression data revealed *AT3G32930* to be a potential functional candidate. Its gene expression difference between infiltrated and control plants is nominally significant at all time points within 24 hours after injection. However, positive control group suggests that only the expression difference at the early stage is significant comparing to the other genes in the genome. This indicates that *AT3G32930* is one of the genes immediately reactive to the injection of *P. syringae* pv. *avrRpm1*, and early stage changes as such potentially triggers chain reactions in the biochemical networks in the plant. As co-expressed genes of AT3G32930 also show similar significant response to the bacteria injection, this thus also suggests a causal role of the detected locus in relevant biological pathways. Furthermore, the Uniprot database^16^ (uniprot.org) showed that the co-expressed genes *STR11, YLMG2, AT1G76405* all function in chloroplasts, which suggest the collapsing mechanism might contain a component of dysfunctional chloroplasts that is linked to a biological pathway related to our detected locus.

Although the protein sequence variations in *AT3G32904* could also be potential functional factors of the detected association signal, recent evidence suggested that cis-regulatory mechanisms are the major contributors to GWAS signals rather than protein coding changes^17^,^18^. Exceptions do exist, such as the stop gained mutation in *CMT2* that we recently reported for temperature adaption^3^. However, in general, cis-regulatory mechanisms provide larger effects on the molecular consequences of mappable loci. This is probably because it is easier for the organism to compensate protein sequence changes than breakdowns of regulatory mechanisms.

Our discovery provides a typical example where multivariate analysis can gain power in association studies, i.e. the statistical significance (chi-squared statistic) increases when the genetic effects on the phenotypes do not agree with the phenotypic correlation level. In our case, the two hypersensitive response traits are so similar to each other on the phenotypic (liability) scale, which is suppose to lead to substantial similarity of genetic effects estimates across the genome. However, the newly detected locus harbors a mutation that has sufficiently different odds ratios on the two traits, which strongly deviates from our null expectation and thus indicates bivariate statistical significance. In our case, a genome-wide significant signal happens to appear for two highly correlated traits, so that such a small sample size can still reveal the signal. With sufficient sample size, lower phenotypic correlation might also be powerful enough to detect other hidden loci associated with complex traits, such as the other pairs of traits analyzed here. Nevertheless, given a limited sample size, such a power boost we see can only be achieved via joint analysis of multiple phenotypes, as the detection of a functional locus for one trait has to borrow information from another correlated trait.

## Materials and Methods

### Single-trait genome-wide association analysis

The bacteria hypersensitive response phenotype data were downloaded from https://github.com/Gregor-Mendel-Institute/atpolydb/blob/master/miscellaneous_data/phenotype_published_raw.tsv, previously published and analyzed by^1^,^10^. The genotype data was downloaded from https://github.com/Gregor-Mendel-Institute/atpolydb/blob/master/250k_snp_data/call_method_75.tar.gz. We imported the data in R using the GenABEL package^19^. To correct for the population structure in these *Arabidopsis thaliana* accessions, single-trait genome scan was performed based on linear mixed models, using the polygenic() and mmscore() procedure in GenABEL. SNPs with MAF < 0.05 were excluded from the analysis. Whole-genome summary statistics including the estimated effects and standard errors from the mmscore() function were stored for subsequent double-trait analyses.

### Double-trait genome-wide association analysis

We performed double-trait genome scans using our recently developed multivariate analysis method implemented in the MultiABEL package^9^. The method takes the whole-genome summary statistics to infer shrinkage phenotypic correlation coefficients and conducts MANOVA analysis. The shrinkage phenotypic correlation coefficient of two traits can be unbiasedly estimated by the correlation of genome-wide Z-scores, which is proportional to the phenotypic correlation on the liability scale, with a shrinkage factor of the square root of sample overlapping proportion. Bivariate p-values are reported. In this way, the bivariate MANOVA analysis is carried out on the liability scale, on partially overlapping sample, without sophisticated bivariate modelling for binary data.

### Locus definition and gene expression analysis

Centered at the top variant of the novel locus, genes within 40 kb distance to the top variant are considered in gene expression analysis. Schmitz *et al*.^13^ data were used to screen for eQTL within the locus. We classified the expression value of those genes into three class and applied eQTL mapping accordingly as have been done by Zan *et al*.^20^. Since it has been shown that measurements on lowly-expressed genes are not reliable in this dataset^20^, we filtered out genes only expressed in less than 50 accession. We applied *qtscore* function from the GenABEL package to quantile transformed expression values for genes expressed in more than 90% accessions and binarized expression values for genes expressed in between 50 and 126 accessions. Significance was declared if any of the SNPs that are in strong LD (r^2^ > 0.6) with our top variant have a p-value below the 5% Bonferroni-corrected significance threshold for the number of tested genes.

Arabidopsis eFP Browser (http://bar.utoronto.ca/efp/cgi-bin/efpWeb.cgi)^14^ was used to extract gene expression change over time under *P. syringae* treatments, where expression means and standard deviations at different time points were extracted. For each gene at each time point after injection, the T-score comparing the mean difference between the bacteria treatment and control plants is calculated as:


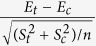


where *E* and *S* are the mean and standard deviation in each experiment group, *t* and *c* stand for treatment and control groups, and n (=3) is the sample size.

### Variants effects prediction

In order to identify plausible functional mutations in the detected locus, we first downloaded the whole-genome sequence data of 1 135*A. thaliana* accessions^4^ of the 1001 Genomes project (http://www.1001genomes.org). We extracted all the sequence variants that had LD r^2^ > 0.50 within 65 kb distance to the top variant. These variants were analyzed by Ensembl variant effect predictor (http://www.ensembl.org/Tools/VEP) for their coding consequence.

## Additional Information

**How to cite this article:** Wang, B. *et al*. Bivariate genomic analysis identifies a hidden locus associated with bacteria hypersensitive response in *Arabidopsis thaliana. Sci. Rep.* **7**, 45281; doi: 10.1038/srep45281 (2017).

**Publisher's note:** Springer Nature remains neutral with regard to jurisdictional claims in published maps and institutional affiliations.
